# Programmable Versus Differential Pressure Ventriculoperitoneal Shunts for Pediatric Hydrocephalus: A 20-Year Single-Center Experience From Saudi Arabia

**DOI:** 10.7759/cureus.43369

**Published:** 2023-08-12

**Authors:** Soha A Alomar, Rothina J Saiedi, Sultan M Albukhari, Majd M Ahmad, Ghaidaa Sindi, Mai Kadi, Saleh S Baeesa

**Affiliations:** 1 Division of Neurosurgery, Department of Surgery, King Abdulaziz University Faculty of Medicine, Jeddah, SAU; 2 Department of Surgery, King Abdulaziz University Hospital, Jeddah, SAU; 3 Department of Community Medicine, King Abdulaziz University, Jeddah, SAU

**Keywords:** shunt revision, shunt complications, programmable shunt valve, ventriculoperitoneal shunt, pediatric hydrocephalus

## Abstract

Background

Shunt malfunction is the most common complication after ventriculoperitoneal shunt (VPS) insertion for pediatric hydrocephalus. The incidence of shunt malfunction and the need for VPS revision may be related to the type of valve used in the shunt. Therefore, we aimed to compare the outcome of VPS in the pediatric age group stratified by differential pressure valves (DPV) and programmable shunt valves (PSV).

Materials and methods

This ethics-approved retrospective study was conducted at a tertiary care hospital in Saudi Arabia. We included 175 children with congenital hydrocephalus who underwent a shunt insertion or revision between 2003 and 2018 and followed them up to December 2022. The VPS complication and revision rates were compared with the patient’s demographics and shunt valve types. The Kaplan-Meier method, log-rank test, and univariate and multivariate Cox proportional hazards regression were used to analyze several variables and subsequent shunt revisions.

Results

Females represented 52% of the study participants, and the mean age of the patients was 21.7 ± 38.4 months. The main indication for VPS was congenital hydrocephalus due to aqueductal stenosis (40%). The differential shunt valve was used in 78.9% and the PSV in 21.1% of the patients. Surgical complications occurred in 33.7% of the patients. Shunt malfunction and infection occurred in 16% and 11.4% of the patients, respectively. The VPS revision rate was significantly lower when PSV was used (odds ratio = 0.39, P < 0.05).

Conclusion

Overall, one-third of the studied pediatric cohort required shunt revision at some point during the 15-year follow-up. However, children with PSV had fewer revision rate-related complications compared to children with DPV during the first five years of follow-up.

## Introduction

Successful management of hydrocephalus using a ventriculoperitoneal shunt (VPS) for cerebrospinal fluid (CSF) was first introduced by Nulsen and Spitz in 1949, who implanted a stainless-steel ball-valve system [1]. The standard shunt valves' function depends on the differential pressure across them, termed the opening pressure. Typically, there are low (0-5 cm of H2O), medium (5-10 cm of H2O), and high pressure (10-15 cm of H2O) shunts for which there are no universal standards. Once open, these valves provide very little resistance to flow [2]. However, an ongoing debate exists regarding the shunt's selection with the correct working pressure and valve type [3-6].

To date, there is no consensus regarding the best shunt valve for pediatric hydrocephalus; nevertheless, there are increasing trends toward using programmable shunt valves (PSV). Selecting a proper shunt valve could minimize flow-related complications that may lead to shunt revision, such as under or over-drainage.

Current studies in the literature remain controversial regarding the difference in VPS outcome between PSV and differential pressure valves (DPV). Therefore, we aimed to evaluate the complication rate of VPS in the pediatric age group stratified by the type of programmable (adjustable) and differential (fixed) pressure valves.

## Materials and methods

Study design

This is an ethics-approved retrospective study of prospectively collected data conducted at King Abdulaziz University Hospital in Jeddah, Saudi Arabia (Biomedical Ethics Research Committee Unit, Faculty of Medicine, King Abdulaziz University; reference number: 14-17).

The electronic records of 175 children with hydrocephalus who underwent VPS insertion between January 2003 and December 2018 were analyzed. Patients aged ≤ 15 years, the legal age of a child in Saudi Arabia, who had VPS inserted for the first time, including those who failed prior other techniques of CSF diversion, including endoscopic third ventriculostomy (ETV), were included. Patients older than 15 years with incomplete data and missing information about the VPS valve type were excluded.

Data collection

After the ethics committee's approval, the hospital's electronic search engine collected medical records. Since this was a retrospective study analyzing medical records, consent from the parents or guardians was not required. The study conformed to the tenets of the Helsinki Declaration of 1975 and its subsequent revisions. Demographic data were collected, and the ventricular size was measured using the radiological evaluation of the ventricle-hemispheric ratio (VHR) pre- and postoperatively. VHR was calculated from the computed tomography (CT) scan films or on ultrasonography by comparing the maximum ventricular diameter of the lateral ventricle's frontal horn at the atrium to the brain's diameter at the same level. The intraoperative opening ventricular pressure measurement was not routinely done in our practice. The patients were also assessed for shunt malfunction, including obstruction, under or over-drainage, and shunt infection. Shunt revision indicates a VPS was inserted at the index or different craniometric points, either frontal pre-coronal (Kocher's) or parietal (Keen's) points, after their previous implant(s) failed, regardless of the underlying cause. Types of the valve, the number of patients who underwent shunt revision, and the time to shunt revision were recorded. The cohort was analyzed based on PSV or DPV types, with the latter also further divided into low and medium DPV.

Statistical analysis

The study analysis was performed using R software version 3.6.3 (R Project, Vienna, Austria). Patients younger than one month were truncated at one month (considered one month old at the time of surgery). Descriptive analysis was performed to assess the relationship between valve type (differential vs. programmable) and demographic and clinicopathological characteristics, using mean (SD) and Student's t-test for continuous variables and proportions and chi-square test for categorical variables. Furthermore, univariate logistic regression was used to assess the association between the valve type and various demographic and clinical factors.

The Kaplan-Meier estimator compared the probability of shunt revision and surgical complications based on the valve type. In addition, the log-rank test was also used to test for equality between the survival curves for surgical complications and shunt revision. The date of VPS insertion was considered the date of the start of the follow-up. The date of the first revision was used for patients in whom shunt malfunctions occurred multiple times. Patients who did not experience the outcomes of interest (complication or revision) were censored at the last follow-up date.

Cox proportional hazards regression was used to assess the factors associated with shunt malfunction and infection. Further survival analysis was performed after five and 10 years to evaluate whether the risk of shunt revision and surgical complications differed according to the VPS type. Hypothesis testing was performed at a 5% level of significance.

## Results

The study included 175 children who underwent VPS for hydrocephalus. Three patients had missing dates of shunt revision and were excluded from the survival analysis. Twenty patients were excluded from the study because they were lost to follow-up or did not follow up on the date scheduled at the time of data collection. Follow-up data from 152 children were completed and used for VPS survival analysis. All shunts used in our cohort were Medtronic™ Strata PSV (Dublin, Ireland) shunts and small or regular DFV sets. A descriptive analysis was performed for all 175 patients. Males and females represented 48% and 52% of the study sample, respectively. The average age of the patients at VPS insertion was 21.7 ± 38.4 months. Congenital hydrocephalus was present in 87.4% of the patients. The main indication for a VPS was aqueduct stenosis (40%). Hydrocephalus with myelomeningocele was the reason for shunting in 18.31% of the patients. A DPV, either low or medium pressure, shunt was used in 78.9%, and PSV in 21.1% of the children. The preoperative and postoperative VHRs were 0.6 ± 0.23 and 0.5 ± 0.21, respectively.

Shunt complications occurred in 33.7% of the patients. The most common surgical complication was a VPS malfunction, which was mostly proximal, occurred in 28 (16%) patients, followed by shunt infection in 20 (11.4%), over-drainage in six (3.43%), and pseudomeningocele at the burr hole site in five patients (2.86%) (Table 1).

**Table 1:** Descriptive characteristics of the study sample

	N = 175	Total
Gender		175
Female	91 (52.0%)	
Male	84 (48.0%)	
Age at surgery (months)	21.7 (38.4)	175
Age category		
≤2 years	51 (29.1%)	
>2 years	124 (70.9%)	
Main indication		175
Aqueduct stenosis	70 (40.0%)	
Chiari malformation	32 (18.31%)	
Dandy-Walker syndrome	20 (11.4%)	
Encephalocele	2 (1.14%)	
Infarction	5 (2.86%)	
Intraventricular hemorrhage-related hydrocephalus	11 (6.29%)	
Meningitis	14 (8.00%)	
Porencephalic cyst	2 (1.14%)	
Schizencephaly	2 (1.14%)	
Traumatic hydrocephalus	1 (0.57%)	
Tumor	16 (9.14%)	
Type of valve		175
Differential pressure	138 (78.9%)	
Programmable	37 (21.1%)	
Type of hydrocephalus		175
Communicating	22 (12.6%)	
Obstructive	153 (87.4%)	
Ventricle hemispheric ratio		
Preoperative	0.60 (0.23)	144
Postoperative	0.50 (0.21)	158
Surgical complications		175
No	116 (66.3%)	
Yes	59 (33.7%)	
Surgical complications		175
No	116 (66.3%)	
Over-drainage	6 (3.43%)	
Pseudomeningocele	5 (2.86%)	
Shunt infection	20 (11.4%)	
Shunt malfunction	28 (16.0%)	
Medical complications		175
No	174 (99.4%)	
Yes	1 (0.6%)	

A logistic regression model detected the significant factors associated with surgical complications. It showed that the only important factor was the type of valve, with programmable valves showing a lower complication rate of 18.9% compared to a 37.7% rate associated with the differential pressure valves, with a 60% reduction in the odds of surgical complications with programmable valves (P = 0.031). A similar result was observed for the need for shunt revision (odds ratio (OR) = 0.43, P < 0.05) (Table 2).

**Table 2:** Factors associated with the odds of surgical complication (logistic regression analysis) Logistic regression analysis showed that programmable valves were associated with a 60% reduction in the odds of surgical complications (p = 0.031). None of the remaining factors showed an association with the incidence of complications.

	No (N = 116)	Yes (N = 59)	Odds ratio	P-value
Gender				
Female	59 (64.8%)	32 (35.2%)	0.87 (0.46; 1.64)	0.677
Male	57 (67.9%)	27 (32.1%)		
Valve				
Differential pressure	86 (62.3%)	52 (37.7%)	0.39 (0.15; 0.92)	0.031
Programmable	30 (81.1%)	7 (18.9%)		
Type of hydrocephalus				
Communicating	16 (72.7%)	6 (27.3%)	1.39 (0.53; 4.14)	0.514
Obstructive	100 (65.4%)	53 (34.6%)		
Ventricle hemispheric ratio				
Preoperative	0.60 (0.45;0.74)	0.60 (0.43;0.73)	0.86 (0.19; 3.92)	0.841
Postoperative	0.47 (0.36;0.62)	0.50 (0.36;0.69)	1.41 (0.29; 6.88)	0.669
Age at surgery, months	7.00 (1.75;27.0)	2.00 (1.00;11.0)	0.99 (0.98; 1.00)	0.165
Cerebral mantle thickness				
	44 (62.9%)	26 (37.1%)		
Normal	6 (60.0%)	4 (40.0%)	1.14 (0.26; 4.50)	0.855
Mild	28 (68.3%)	13 (31.7%)	0.79 (0.34; 1.78)	0.574
Moderate	38 (70.4%)	16 (29.6%)	0.72 (0.33; 1.53)	0.390
Entry site				
Frontal	19 (70.4%)	8 (29.6%)	0.48 (0.13; 1.74)	0.266
Parietal	88 (67.2%)	43 (32.8%)	0.55 (0.19; 1.59)	0.264

Regarding the shunt infection, cause-specific Cox regression analysis showed that the occipital (hazard ratio (HR) = 0.20, P = 0.05) and parietal (HR = 0.28, P = 0.02) entry sites were associated with a lower hazard of acquiring a shunt infection (Table 3).

**Table 3:** Cause-specific analysis for shunt malfunction and infection

	Shunt infection		Shunt malfunction	
	Hazard ratio	P-value	Hazard ratio	P-value
Gender				
Female	0.953	0.919	1.865	0.145
Male				
Valve				
Differential pressure	1.31	0.633	0.41	0.229
Programmable				
Type of hydrocephalus:				
Communicating	1.44	0.63	1.25	0.72
Obstructive				
Ventricle hemispheric ratio				
Preoperative	1.10	0.939	2.94	0.279
Postoperative	1.15	0.903	3.08	0.291
Age at surgery (months)	0.99	0.642	0.99	0.291
Cerebral mantle thickness				
Mild	2.48	0.417	0	0.998
Moderate	2.36	0.172	0.41	0.094
Normal	1.98	0.29	0.58	0.282
Entry site				
Frontal	0.20	0.05	0.88	0.88
Parietal	0.28	0.02	0.95	0.946

The shunt revision rate was higher when a DPV was used (40.4% vs. 22.2%, P = 0.043). When stratifying the analysis by age, results showed that the incidence of surgical complications was significantly lower when PSV was used (OR = 0.24, P < 0.05) in patients older than two years of age during the first five years of follow-up. A non-significant difference was observed in patients younger than two years (OR = 1.61, P > 0.05). Age, sex, type of hydrocephalus, and VHR were not associated with an increased surgical complication, shunt revision, or infection rate. The duration after surgical complications occurred and revision was needed was slightly longer with the PSV (49.7 vs. 47.8 days and 52.3 vs. 46.9, respectively). However, it was not statistically significant (P = 0.08 and 0.106, respectively). In addition, there was a higher rate of over-drainage in DPV than in PSV (7.25% vs. 2.7%), but this difference was not statistically significant (P = 0.348) (Table 4).

**Table 4:** Comparison of valve types in terms of complication rate, shunt revision rate, age, gender, and ventricle hemispheric ratio pre and postoperatively among all cases (n = 175)

	Differential pressure (N = 138)	Programmable (N = 37)	Odds ratio	P-value
Gender				
Female	68 (49.3%)	23 (62.2%)	0.60 (0.28; 1.25)	0.170
Male	70 (50.7%)	14 (37.8%)		
Type of hydrocephalus				
Communicating	18 (13.0%)	4 (10.8%)	1.20 (0.41; 4.52)	0.752
Obstructive	120 (87.0%)	33 (89.2%)		
Ventricle hemispheric ratio				
Preoperative	0.59 (0.25)	0.60 (0.17)	1.09 (0.21; 5.62)	0.919
Postoperative	0.50 (0.21)	0.49 (0.22)	0.76 (0.12; 4.85)	0.776
Age at surgery (months)	22.9 (41.2)	17.4 (26.0)	1.00 (0.98; 1.01)	0.439
Age category				
<2	44 (31.9%)	7 (18.9%)	1.97 (0.84; 5.26)	0.125
>2	94 (68.1%)	30 (81.1%)		
Surgical complications				
No	86 (62.3%)	30 (81.1%)	0.39 (0.15; 0.92)	0.031
Yes	52 (37.7%)	7 (18.9%)		
Shunt revision				
No	81 (59.6%)	28 (77.8%)	0.43 (0.17; 0.98)	0.043
Yes	55 (40.4%)	8 (22.2%)		
Time to surgical complication (n = 56)	28.8 (47.8)	33.8 (49.7)	1 (0.99; 1.02)	0.080
Time to shunt revision (n = 60)	29.6 (46.9)	42.6 (52.3)	1.01 (0.99; 1.02)	0.106
Cerebral mantle thickness				
	58 (42.0%)	12 (32.4%)		
Normal	6 (4.35%)	4 (10.8%)	3.19 (0.69; 13.4)	0.130
Mild	34 (24.6%)	7 (18.9%)	1.00 (0.34; 2.78)	0.996
Moderate	40 (29.0%)	14 (37.8%)	1.68 (0.70; 4.11)	0.245
Catheter entry site				
Frontal	14 (10.1%)	3 (8.11%)		
Occipital	21 (15.2%)	6 (16.2%)	1.30 (0.28; 7.44)	0.744
Parietal	103 (74.6%)	28 (75.7%)	1.22 (0.36; 5.85)	0.765
Over drainage				
No	128 (92.8%)	36 (97.3%)	0.4 (0.02; 2.24)	0.348
Yes	10 (7.25%)	1 (2.7%)		
Stratified analysis				
Surgical complications (age ≤ 2 years)				
No	30 (68.2%)	4 (57.1%)	1.61 (0.27; 8.72)	0.586
Yes	14 (31.8%)	3 (42.9%)		
Surgical complications (age > 2 years)				
No	56 (59.6%)	26 (86.7%)	0.24 (0.06; 0.67)	0.005
Yes	38 (40.4%)	4 (13.3%)		

The majority of DPV was medium pressure (121 patients); only 20 patients had low-pressure valves. In addition, there was no significant difference between the medium and low-pressure valve groups regarding age, sex, type of hydrocephalus, VHR, surgical complications, shunt revision, or over-drainage (Table 5).

**Table 5:** Comparison of differential pressure valve recipients in terms of complication rate, shunt revision rate, age, gender, and ventricle hemispheric ratio pre and postoperatively (n = 141)

	Low (N = 20)	Medium (N = 121)	Odds ratio	P-value
Gender				
Female	11 (55.0%)	58 (47.9%)	1.32 (0.50; 3.54)	0.570
Male	9 (45.0%)	63 (52.1%)		
Type of hydrocephalus				
Communicating	4 (20.0%)	13 (10.7%)	2.11 (0.52; 6.96)	0.271
Obstructive	16 (80.0%)	108 (89.3%)		
Ventricle hemispheric ratio				
Preoperative	0.62 (0.21)	0.59 (0.25)	0.62 (0.07; 5.39)	0.667
Postoperative	0.57 (0.22)	0.49 (0.21)	0.16 (0.02; 1.69)	0.128
Age at surgery (months)	11.2 (24.2)	24.6 (42.4)	1.01 (0.99; 1.03)	0.194
Age category				
<2	8 (40.0%)	37 (30.6%)	1.52 (0.55; 4.03)	0.414
>2	12 (60.0%)	84 (69.4%)		
Surgical complications				
No	11 (55.0%)	77 (63.6%)	0.70 (0.27; 1.88)	0.470
Yes	9 (45.0%)	44 (36.4%)		
Shunt revision				
No	11 (55.0%)	72 (60.5%)	0.80 (0.30; 2.15)	0.647
Yes	9 (45.0%)	47 (39.5%)		
Time to surgical complication (n = 53)	31.8 (64.4)	28.7 (44.3)	1 (0.98; 1.02)	0.868
Time to shunt revision (n = 50)	31.8 (64.4)	27.7 (45.1)	1 (0.98; 1.01)	0.877
Cerebral mantle thickness				
Normal	2 (10.0%)	7 (5.79%)	0.56 (0.10; 4.68)	0.541
Mild	4 (20.0%)	30 (24.8%)	1.20 (0.34; 5.01)	0.782
Moderate	6 (30.0%)	35 (28.9%)	0.95 (0.30; 3.18)	0.928
Catheter entry site				
Frontal	4 (20.0%)	17 (14.0%)	0.37 (0.01; 3.05)	0.383
Parietal	15 (75.0%)	91 (75.2%)	0.53 (0.02; 3.00)	0.530
Over drainage				
No	17 (85.0%)	114 (94.2%)	0.34 (0.08; 1.81)	0.188
Yes	3 (15.0%)	7 (5.79%)		

**Figure 1:**
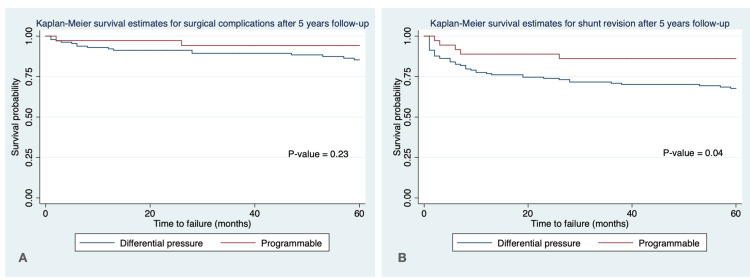
Kaplan-Meier survival curve for surgical complications (A) and shunt revision (B) after five years of follow-up

**Figure 2:**
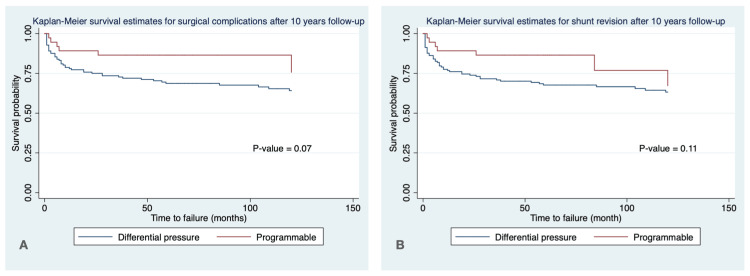
Kaplan-Meier survival curve for surgical complications (A) and shunt revision after 10 years of follow-up

Figures 1, 2 show the Kaplan-Meier survival estimates for surgical complications and shunt revision after five years (Figure 1) and 10 years (Figure 2) of follow-up. The probability of shunt revision was significantly lower in the PSV type compared to the DVP type at five years of follow-up (p-value = 0.04).

There was no statistically significant difference between the surgical complications at five years and 10 years of follow-up and shunt revision at 10 years of follow-up between PSV and DVP types (p-value > 0.05).

## Discussion

Despite the overwhelming evidence that CSF diversion with VPS placement has been the cornerstone of pediatric and adult hydrocephalus management, this procedure still has a considerably high complication and failure rate. In addition, among hydrocephalus etiologies, obstructive hydrocephalus in the pediatric age group is the most common [2].

Complication rates remain extremely high [7]. Approximately 11-25% of the shunt failures occurred within six to 12 months of shunt placement [2,8]. Most sources have reported a significantly higher number of shunt revisions and replacements in the pediatric age group than in adults. In a retrospective analysis of 64 pediatric patients followed over 15 years, only 15.5% did not require a revision during the follow-up period, and 17.3% required three or more revisions [9].

Different shunt models aim to lower complication rates and increase durability. Two types of valves are available: differential (fixed or flow) and programmable (adjustable) valves. Any of these devices can be used with or without the anti-siphon device. Anti-siphon devices minimize the change in drainage volume with sudden changes in position.

A retrospective analysis by Robinson et al., including 200 patients less than one-year-old, concluded that the medium-pressure valve was better than the low-pressure valve. The low-pressure valve allows more CSF drainage, leading to smaller ventricles with an increased predisposition to proximal occlusion [10].

Drake et al. studied 344 pediatric patients randomized to receive either a fixed pressure valve with the anti-siphon device (Delta Valve, Medtronic PS Medical, Goleta, CA), a fixed flow valve with the anti-siphon device (Orbis-Sigma Valve, Cordis, Miami, FL), or a fixed pressure valve without the anti-siphon device. There were no significant differences between the three groups regarding shunt failure (27% for the Orbis-Sigma, 33% for the Delta Valve, and 34% for the valve without an anti-siphon device) [5].

One of the reported shunt complications is under- or over-drainage. Complications of over-drainage include subdural hematoma (SDH) or hygroma, slit ventricles, and low-pressure headaches. Under-drainage results in persistent ventriculomegaly and large head size.

Pollack et al. compared 377 pediatric patients randomized to receive either an adjustable or fixed pressure valve. The two groups did not differ in reoperation rate, subdural hygromas, SDH incidence, or under-drainage [11].

A study conducted at Johns Hopkins Hospital, including 279 pediatric patients with hydrocephalus, showed that the rate of overall shunt revision, as well as the rate of proximal obstruction, was higher for DPV compared to that for PSV (54% vs. 35% and 28% vs. 12%, respectively). However, there was no difference in distal obstruction, infection, or disconnection between the two types of valves [12]. In our study, the cause of shunt obstruction, either proximal or distal, was not documented in many cases; thus, we could not confirm whether PSV had a lower proximal obstruction rate than DPV.

A meta-analysis of 11 studies with a total of 2622 patients (pediatric and adult) was conducted in 2017 regarding PSV safety and efficacy. No difference in the one-year shunt survival rate between PSV and DPV was found. Additionally, infection and catheter-related complication rates were similar among the two groups. However, PSV was associated with a significantly reduced revision rate (relative risk (RR), 0.56; 95% confidence interval (CI), 0.45-0.69) and over- or under-drainage complication rate (RR, 0.55; 95% CI, 0.32-0.96) among observational studies, especially in pediatric patients aged 18 years or less [13].

A randomized prospective study of 40 pediatric patients in 2012 by Sinha et al. demonstrated no statistically significant difference in the outcome when either the low- or medium-pressure valves were used. The degree of resolution of dilated ventricles was also independent of shunt opening pressure. In addition, there was no difference in complication rates with either valve type [14].

A randomized trial of PSV compared with conventional DPV published by Carmel et al. concluded that there was no significant difference in the incidence of subdural fluid collections between the PSV and DPV treatment groups. However, the PSV feature provided a considerable advantage in treating subdural collections [15].

Our study reported over-drainage complications in the form of subdural collection for two DPV and one PSV recipient. The DPV recipients treated this by changing to PSV.

In our cohort, the PSV group had a lower rate of surgical complications and shunt revisions. In general, over- and under-drainage are frequent problems, and these complications can be avoided with the help of an adjustable shunt. However, adjustable shunts may appear to be more expensive. In addition, the possibility of spontaneous pressure changes and frequent checks and adjustments are limitations to their use. Yet, this difference is significantly reduced when the cost of complications and surgeries is considered.

The risk of infection was lower at the occipital and parietal entry points than at the frontal one. One explanation could be that the frontal entry point requires another incision along the shunt pathway for tunneling, which may predispose the shunt tubing to a higher risk of infection.

Wu et al. found that the strongest independent risk factor for VPS complications was the type of hydrocephalus, with a significantly greater risk associated with congenital and obstructive hydrocephalus compared to non-communicating hydrocephalus. Additionally, male sex and low socioeconomic status were associated with an increased risk of shunt complications [16].

In our study, the type of hydrocephalus and sex did not affect the infection, the overall complication, or the shunt revision rates. Further prospective studies, preferably randomized control trials, are needed, as this issue remains unresolved in the literature.

Study limitations

This is a retrospective study with limitations related to the available data in medical records. Another limitation is that pediatric patients from different age groups, from neonates to 15-year-olds, were included. Before the closure of the fontanelles and fusion of the sutures, infants may be more prone to under- or over-drainage complications; thus, this age group should be studied as a separate entity to reach a firm conclusion. Factors such as the patient's age and incomplete data could affect this study's results.

In addition, a significant proportion of the patients who were either excluded due to missing data or lost to follow-up may have skewed our study results. Additionally, the valve is only one part of the shunt, and there may be many factors other than the type of valve that might predispose the patient to shunt complications. Despite the abovementioned shortcomings, this study contributes substantially to the scientific pool of knowledge. This study analyzed the pediatric population's detailed data concerning the valve type in our region with hydrocephalus undergoing VPS placement.

## Conclusions

Nearly one-third of the studied cohort required a shunt revision at some point during the follow-up. Patients with PSV had significantly lower revision rates than those with DPV during the first five years of follow-up. However, longer follow-ups revealed no statistical difference in the outcome between the two types of shunts in pediatric hydrocephalus.
